# Modern Sociology

**Published:** 1903-02-21

**Authors:** 


### Modern Sociology.

### WHERE OUR LOAVES ARE MADE.

Our bakers are not happy at present. According to the present Factory and Workshops Act an underground bakehouse cannot be used after January 1st, 1904, unless it is certified by the District Council of the neighbourhood where it stands, as being "suitable as regards construction, light, ventilation, and in all other respects." In face of this formidable, but rather vague warning, the bakers want to have some definite notion what the District Council might reasonably consider to be suitable. The Medical Officers of Health throughout the country are very willing to oblige the bakers by telling them what they—the Medical Officers of Health—consider to be the minimum requirements for underground bakehouses—if underground bakehouses there needs must be. Whether the baking trade and the sanitarians will come to an understanding is, however, doubtful, and a proposed conference between representatives of the National Association of Master Bakers and Confectioners of Great Britain and representatives of the Incorporated Society of Medical Officers of Health has in the meantime been postponed at the request of the bakers.

That some improvement in the conditions of labour in underground bakehouses is necessary, in the interest both of the workers in them and the people who eat the bread, can hardly be doubted. Dr. Newman, the Medical Officer of Health for the Borough of Finsbury, has made some investigations of the underground bakehouses in his district which show that their condition leaves, as a rule, much to be desired. All are not equally bad, and every inch which the bakehouse rises above the level of the ground is an advantage, but some are altogether cellars, or, as Dr. Newman calls them, "wells," and there the ventilation is defective, and consequently the air is bad, and bacteria flourish exceedingly. Carbonic acid gas is present in these bakehouses in great excess of the normal amount of even the London atmosphere. The average proportion is four volumes of carbonic acid for every 10,000 volumes of air. In Finsbury the proportion in the street air, as estimated by an analyst, is slightly higher, 4·3, and in the air of an above-ground bakehouse, 4·9. But in underground bakehouses it varied from 12 to 17·5 per 10,000. The fermentation of the dough would account for a certain degree of this vitiation—and therefore accentuates the need for exceptionally good ventilation—but in addition, there is the amount [illegible]oduced by the respiration of the workers, about a cubic [illegible] of carbonic acid per hour, and that produced by the [illegible]ing of gas as an illuminant, which is estimated at from [illegible] 5 cubic feet per hour for every ordinary jet. Incan[illegible]nt lights are much less mischievous, and electric light [illegible]tter still. But the ordinary gas jet is still frequently [illegible]d.

[illegible] test for the presence of bacteria, Dr. Newman exposed [illegible]e various bakehouses he examined, three agar plates for [illegible] minutes, and then incubated these at blood heat for [illegible]ours. The result showed that whereas on the 9·6-inch [illegible]tes exposed in underground bakehouses, the number of [illegible]teria varied from 600 to 800; a plate exposed in a bake[illegible]se above ground showed only 200. This sufficiently [illegible]ws that the air of underground bakehouses is far from [illegible]sfactory. It is difficult to say to what extent it affects [illegible] health of the workers, for the vital statistics available [illegible]e for all bakers, without distinction as to the kind of shop they work in. Such as they are, however, the figures show that the deaths among bakers from rheumatic fever, diabetes, and urinary diseases are above the average for occupied males generally. Dr. Newman found that 54 per cent. of the deaths of persons working in bakehouses recorded during the last five years were due to diseases of the lungs. This would seem to imply a heavy indictment against the polluted atmosphere to be found in these places, all the more that a baker's work does not expose him to many unfavourable conditions which injure other workers. The dryness of the bakehouse tends rather to prevent the development of lung diseases, but on the other hand there may be a special danger to him from going from a hot, dry atmosphere into one which is cold and damp when he leaves his work.

With regard to the consumers, there are no statistics available to prove that persons have suffered from eating bread baked in underground bakehouses, but the public as a whole is now sufficiently fastidious to dislike the idea of food coming from places so insanitary. If the worker profits by any change that may be made in these workshops, the consumer will feel that the benefit is more or less his also.

The suggestions made by the Society of Medical Officers of Health will, if carried out, greatly improve the conditions of underground bakehouses — indeed, they will entirely prevent their being entirely below the surface of the ground, for clause 7 of the proposed regulations asks that: "The underground bakehouse shall be adequately lighted with daylight throughout to the satisfaction of the sanitary authority, and the lighting maintained shall be such that the Official Abstract of the Factory and Workshop Act, 1901, may be ordinarily read in all parts of the bakehouse between the hours of 11 A.M. and 3 P.M." For the rest the height of the bakehouse walls is to be not less than 8 feet, or in large places where the floor area exceeds 300 square feet, at least 8 feet 6 inches. The walls are to be composed of some material which is "hard, smooth, durable, and impermeable to damp." It is demanded that the floor and ceiling fulfil similar conditions. No outside staircase is to terminate directly within an underground bakehouse, and any opening into the shop above is to be so covered as to prevent the entrance of dust. With regard to ventilation it is desired that "fresh clean air," shall be supplied in such quantity as to provide not less than 3,000 cubic feet of air per hour for each person employed, with any additional amount required for the purposes of combustion. Where mechanical power is needed to provide for the supply of air, "the fresh air shall be taken from a height above the level of the adjoining ground of not less than six feet, and be distributed to different parts of the underground bakehouse in such a manner as to change the air of such bakehouse in all parts." There are also suggestions offered for the provision of lavatories, etc., outside the bakehouse for the depositing of wearing apparel and the storage of flour outside the place. If all these regulations were carried into effect there is no doubt that the objections to the underground bakehouse would largely cease. Unfortunately, the fact that the decision as to whether or not the bakehouse is satisfactory in all respects is to rest with the local authorities makes one rather doubtful if all necessary improvements will be carried out. If each bakehouse is to be judged, not according to the strict letter of a strict law, but according to its individual merits in the eyes of the district council, there may be less improvement than one could desire. The merits of a workshop are apt to be affected, in local eyes, by the merits and influence of its owner, and if reform is to be achieved, it will be well to allow but little scope for the action of local opinion.

